# Glucocorticoids Protect Neonatal Rat Brain in Model of Hypoxic-Ischemic Encephalopathy (HIE)

**DOI:** 10.3390/ijms18010017

**Published:** 2016-12-22

**Authors:** Benjamin Harding, Katherine Conception, Yong Li, Lubo Zhang

**Affiliations:** 1 Division of Neonatology, Department of Pediatrics, Loma Linda University Children’s Hospital, Loma Linda, CA 92354, USA; bharding@llu.edu; 2 Center for Perinatal Biology, Loma Linda University School of Medicine, Loma Linda, CA 92350, USA; kconcepcion@llu.edu (K.C.); yoli@llu.edu (Y.L.)

**Keywords:** hydrocortisone, dexamethasone, lipopolysaccharides, intranasal, neuroprotection, Rice-Vannucci, neonatal, hypoxic-ischemic, encephalopathy

## Abstract

Hypoxic-ischemic encephalopathy (HIE) resulting from asphyxia in the peripartum period is the most common cause of neonatal brain damage and can result in significant neurologic sequelae, including cerebral palsy. Currently therapeutic hypothermia is the only accepted treatment in addition to supportive care for infants with HIE, however, many additional neuroprotective therapies have been investigated. Of these, glucocorticoids have previously been shown to have neuroprotective effects. HIE is also frequently compounded by infectious inflammatory processes (sepsis) and as such, the infants may be more amenable to treatment with an anti-inflammatory agent. Thus, the present study investigated dexamethasone and hydrocortisone treatment given after hypoxic-ischemic (HI) insult in neonatal rats via intracerebroventricular (ICV) injection and intranasal administration. In addition, we examined the effects of hydrocortisone treatment in HIE after lipopolysaccharide (LPS) sensitization in a model of HIE and sepsis. We found that dexamethasone significantly reduced rat brain infarction size when given after HI treatment via ICV injection; however it did not demonstrate any neuroprotective effects when given intranasally. Hydrocortisone after HI insult also significantly reduced brain infarction size when given via ICV injection; and the intranasal administration showed to be protective of brain injury in male rats at a dose of 300 µg. LPS sensitization did significantly increase the brain infarction size compared to controls, and hydrocortisone treatment after LPS sensitization showed a significant decrease in brain infarction size when given via ICV injection, as well as intranasal administration in both genders at a dose of 300 µg. To conclude, these results show that glucocorticoids have significant neuroprotective effects when given after HI injury and that these effects may be even more pronounced when given in circumstances of additional inflammatory injury, such as neonatal sepsis.

## 1. Introduction

Worldwide, intrapartum-related events or birth asphyxia, which results in hypoxic-ischemic encephalopathy (HIE), is the leading cause of death among term infants [1]. HIE is also the most common cause of neonatal brain damage that can result in severe long term neurologic morbidity such as seizures, developmental delay, and cerebral palsy in up to 25% of survivors [2]. In addition, the role of neonatal infection and inflammation on HIE severity is becoming more apparent as clinical research has shown an association with maternal peripartum infection (chorioamnionitis), elevated neonatal cytokines, and worse neurologic outcomes associated with HIE [3].

Unfortunately, despite the large morbidity and mortality associated with HIE, currently, the only approved and validated treatment in addition to supportive care for infants with HIE is therapeutic hypothermia (whole body cooling). This therapy requires specialized centers which limits its availability and its use is dependent on specific criteria, such as gestational age, injury severity and, most importantly, time from the event to the initiation of treatment (must be initiated within 6 h from the time of the event) [4].

Additional therapeutic agents have been proposed and investigated throughout the years, including glucocorticoids, such as dexamethasone. Interestingly, use of dexamethasone has been shown in previous animal models to have both neuroprotective and neurotoxic effects depending on its dose and timing and these conflicting results have resulted in its disregard as a serious potential clinical therapeutic option in the past [5,6,7]. However, recent evidence has continued to demonstrate the neuroprotective effects of dexamethasone when given prior to a hypoxic-ischemic (HI) injury in the neonatal rat model through various biological mechanisms such as the up-regulation of vascular endothelial growth factor (VEGF) and Prostaglandin D2 (PGD2) synthesis via the glucocorticoid receptor (GR) [8,9]. One potential reason for the improved success of dexamethasone in the more recent studies may be its method of direct brain delivery via intracerebroventricular (ICV) injection with lower doses rather than the traditional delivery method via intraperitoneal (IP) injection with higher doses, which inherently results in more systemic effects and side effects.

Clinically, prophylactic interventions for HIE would not be practical due to its relatively low incidence and the acute nature of HI events. Therefore, any therapeutic intervention for HIE must demonstrate neuroprotective effects when given in a certain period of time after an HI injury. Additionally, delivery by direct brain injection in neonates would be a significantly invasive procedure and would be preferentially avoided if at all possible. In a recent study by our lab, treatment with the microRNA-210 inhibitor (promotes up-regulation of GR expression) was given 4 h post HI-injury in neonatal rats via both direct ICV injection as well as a novel method of intranasal instillation [10]. A significant reduction in brain injury was demonstrated with both methods of delivery suggesting that both post-insult treatment and direct delivery without brain injection targeting GR in the brain are feasible therapeutic options.

The present study tested two glucocorticoids, dexamethasone, and hydrocortisone, as post-injury HI therapies by both direct delivery (ICV) as well as intranasal administration using a modified version of the well-established Rice-Vannucci rat model of HIE [11]. The Rice-Vannucci neonatal rat model is the most commonly used animal model of hypoxic-ischemic encephalopathy (HIE) that caused by systemic asphyxia, and main advantage of the rodent model is the extent of its characterization over the years. As neonatal sepsis is frequently seen in compounding HIE injury, a novel rat model of HIE combined with LPS sensitization was established and tested for the therapeutic potential of hydrocortisone treatment to study the effects of additional inflammation caused by neonatal sepsis that is frequently seen in compounding HIE injury. When compared to rats with HI injury alone, the LPS sensitized rats showed significantly increased brain infarction size.

In all three experimental protocols brain infarction size was analyzed and compared between control and steroid treatment groups. The results demonstrated neuroprotective effects of dexamethasone when given by ICV injection after HI injury. Additionally, when hydrocortisone was given both by ICV injection and intranasal instillation in both the HI and HI with sepsis models, it also demonstrated significant neuroprotection.

## 2. Results

As stated above, our study examined both dexamethasone and hydrocortisone treatments via ICV injection and intranasal administration after HI injury in a neonatal rat model of HIE. In addition, a neonatal rat model of HIE and sepsis was established and tested with hydrocortisone treatment via both ICV and intranasal delivery methods.

### 2.1. Dexamethasone after HI Injury via ICV Injection and Intranasal Administration

Since previous studies involving glucocorticoids and neuroprotection focused on dexamethasone given as a pre-treatment prior to HI injury, our initial experiment examined if dexamethasone was neuroprotective when given in a short window after an HI injury. ICV injection was tested first to determine if direct delivery would be effective, and then the study was repeated with the non-invasive intranasal dexamethasone administration at varying doses.

**Figure 1:**
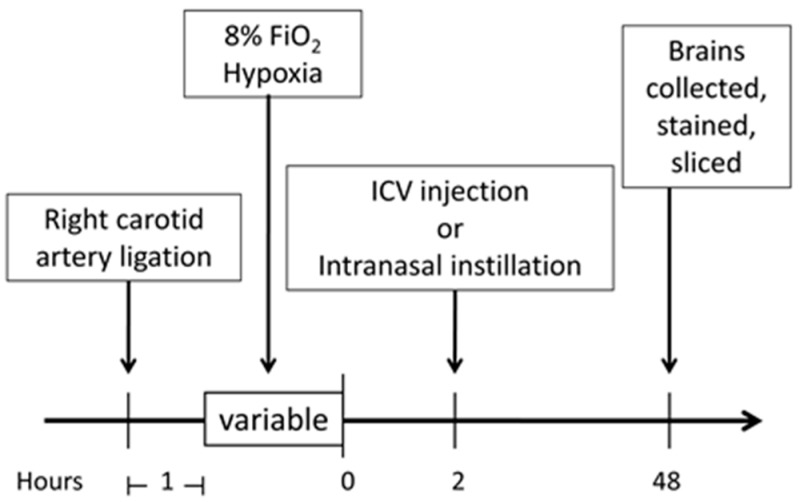
Experimental protocol timeline. Outline of the experimental timeline is described in the materials and methods section. The hypoxia time was varied slightly depending on the experimental groups (dexamethasone, hydrocortisone, and lipopolysaccharide (LPS) models) and are detailed in each respective methods section. ICV, intracerebroventricular.

**Figure 2:**
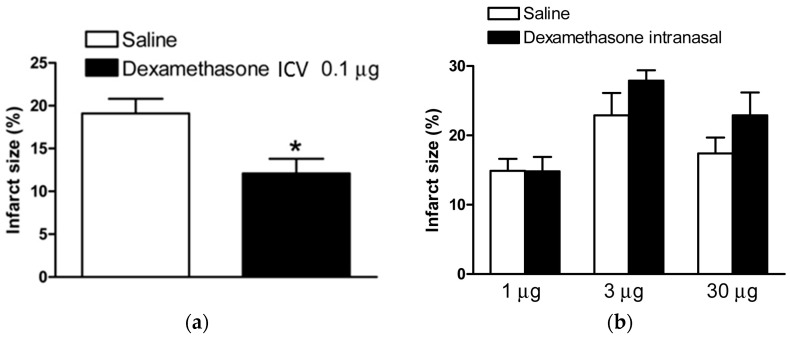
Dexamethasone treatment after HI injury. Dexamethasone via ICV injection or intranasal instillation administered 2 h after HIE insult. (**a**) Dexamethasone, 0.1 µg, (*n* = 18) compared to saline control (*n* = 20) via ICV injection significantly reduced brain infarct size in rat pups; and (**b**) no significant difference was seen when comparing dexamethasone, 1 µg, (*n* = 14) vs. saline (*n* = 17), 3 µg (*n* = 8) vs. saline (*n* = 8), or 30 µg (*n* = 10) vs. saline (*n* = 12) via intranasal administration. Data are means ± SEM, * *p* < 0.05.

Dexamethasone (0.1 µg) or a saline control was given via ICV injection to P7 Sprague Dawley rat pups following right carotid artery ligation and 8% hypoxia treatment as described in the materials and method section below. The rat brains were then collected, sliced, and stained 48 h following the HI insult and the brain infarction size was measured and compared to control rats with HI injury and ICV saline (placebo). The treatment protocol is shown in Figure 1. The mean brain infarction size for the dexamethasone treated rats was 12.4% ± 1.7% vs. 19.4% ± 1.7% for the saline control rats (*p* < 0.05), Figure 2a. These findings demonstrated that dexamethasone significantly decreased overall brain infarction and provided neuroprotection when given after an HI injury by direct brain administration.

In the next experiment, the same HI injury was performed and pups were then randomized to receive intranasal dexamethasone or saline. The rats were divided into three dexamethasone dosing groups (1 µg, 3 µg, and 30 µg) and compared against control rats from within the same litter groups. No significant difference was seen when comparing the dexamethasone and control groups at any of the tested doses (*p* > 0.05), Figure 2b, and, therefore, these findings did not show any neuroprotection from intranasal dexamethasone when given after HI injury.

### 2.2. Hydrocortisone after HI Injury via ICV Injection and Intranasal Administration

**Figure 3:**
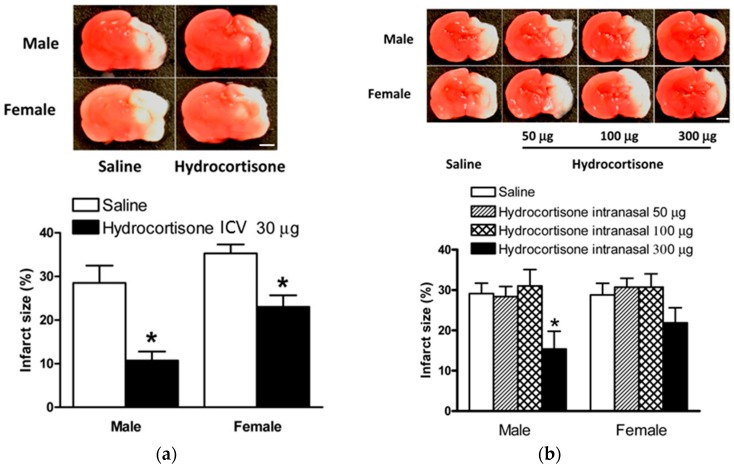
Hydrocortisone treatment after HIE injury. Hydrocortisone via ICV injection or intranasal instillation administered 2 h after HIE insult. Representative brain slices are shown above. (**a**) Hydrocortisone, 10 µg, (*n* = 10) vs. saline (*n* = 11) via ICV injection significantly reduced brain infarct size in both male and female rat pups; (**b**) hydrocortisone, 300 µg, (*n* = 11), but not 50 µg (*n* = 10) or 100 µg, vs. saline (*n* = 17) via intranasal administration significantly reduced brain infarct size in male rat pups. Data are means ± SEM, * *p* < 0.05. Scale bars, 2 mm.

As stated earlier, there is some evidence that neonatal dexamethasone treatment for other clinical conditions may have negative long term neurodevelopmental effects when given to infants systemically depending on the timing and dose [12]. Therefore, we also investigated the potential neuroprotective effects of hydrocortisone post-treatment on neonatal HIE by both ICV and intranasal administration. As shown in Figure 3a, hydrocortisone (10 µg) after HI injury via ICV injection compared to saline conferred neuroprotection in both male and female rat pups subjected to HI injury (10.7% ± 2.1% vs. 28.5% ± 4.0% for males; 23.0% ± 2.7% vs. 35.3% ± 2.0% for females, *p* < 0.05), respectively.

Hydrocortisone was then given after HI injury via intranasal administration in three dosing groups (50 µg, 100 µg, and 300 µg). No difference from the control was seen at the lower doses of 50 µg or 100 µg, however, when the dose was increased up to 300 µg it was found to significantly attenuate brain injury in the male rat pups after HIE insult compared to saline (15.4% ± 4.3% vs. 29.1% ± 2.6%, *p* < 0.05), Figure 3b. In addition, although it did not reach statistical significance, we also observed a substantial reduction in brain infarct size in the hydrocortisone 300 µg treated female rat pups (22.0% ± 3.7% vs. 28.8% ± 3.9%), Figure 3b.

### 2.3. Model of LPS Sensitization in Neonatal Rat Brains with HIE

**Figure 4:**
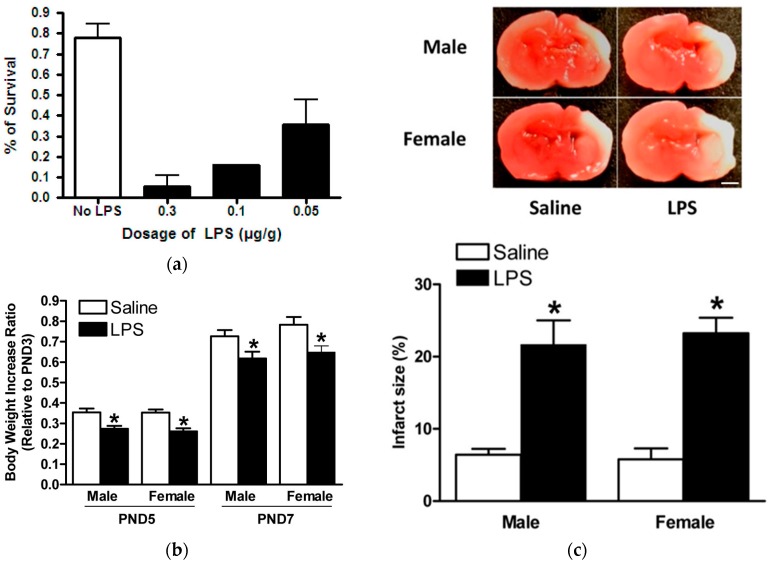
LPS sensitizes neonatal rat brain to HIE. LPS intraperitoneal (i.p.) injection prior to HIE insult on postnatal day 7 (PND7). (**a**) Different doses of LPS via i.p. injection administered 4 h before HIE significantly increased mortality rate in rat pups, compared with the saline control; (**b**) LPS, 0.05 mg/kg, (*n* = 26) via i.p. injection on PND3 and PND5 significantly hindered normal body weight increases in rat pups compared to controls (*n* = 22); and (**c**) representative brain slices are shown above. Pups given LPS, 0.05 mg/kg, (*n* = 23) via i.p. injection on PND3 and PND5 had significantly increased brain infarct size in both genders of neonatal rat pups after HIE insult, compared to controls (*n* = 18). Data are means ± SEM, * *p* < 0.05. Scale bar, 2 mm.

As mentioned previously, it would be expected, and there is now growing evidence to support, that systemic infection and inflammation may exaggerate brain injury in response to neonatal HIE [3]. Therefore, we evaluated the possible adverse effects of acute and chronic infections on neonatal HIE via a series of LPS challenge procedures in rat pups. We found that the acute LPS challenge 4 h prior to HIE induced a dose-dependent decrease in the survival rate in neonatal rats subjected to HI injury, Figure 4a. Therefore, it was not feasible to employ this acutely treated model to continue further mechanistic investigations because of its high mortality and limited available samples.

Interestingly, the chronic LPS challenge model resulted in much lower mortality, which was comparable to our previously-conducted neonatal HIE model (<5%). In addition, we showed that chronic LPS challenge adversely hindered rat pup growth development, which presented as significantly diminished body weight increases in both male and female rat pups, Figure 4b. Most importantly, the chronic LPS challenge significantly increased brain infarct size after HI injury in both male and female neonatal rat pups compared to the controls (21.6% ± 3.4% vs. 6.4% ± 0.8% for males; 23.2% ± 2.2% vs. 5.8% ± 1.5% for females, *p* < 0.05) respectively, Figure 4c. This feasible chronic LPS model with a confirmed detrimental role of systemic infection in neonatal brain HI injury then served as the model for treatment with hydrocortisone in the novel model of HI injury and sepsis.

### 2.4. Hydrocortisone via ICV Injection and Intranasal Administration in Model of HIE with LPS Sensitization

**Figure 5:**
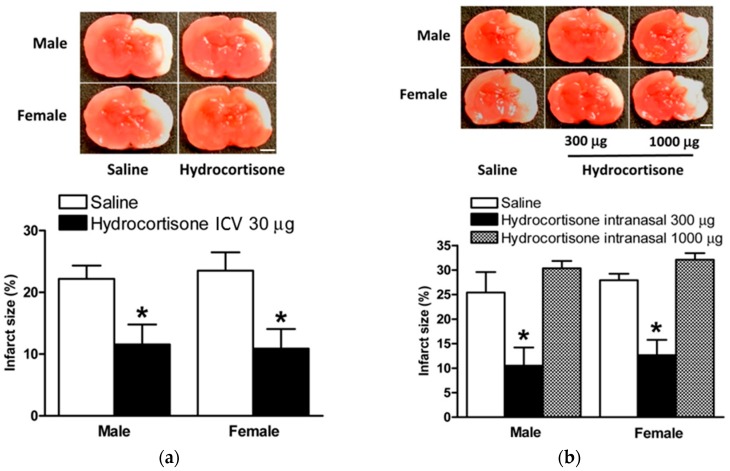
Hydrocortisone protects neonatal rat brain after LPS sensitization and HIE. LPS 0.05 mg/kg via i.p. injection administered on postnatal day 3 (PND3) and 5 (PND5) with HI injury conducted on PND7. Hydrocortisone via ICV injection or intranasal instillation administered 2 h after HIE insult. Representative brain slices are shown above. (**a**) Hydrocortisone, 30 µg, (*n* = 16) via ICV injection significantly reduced brain infarct size in both male and female rat pups vs. controls (*n* = 22); and (**b**) hydrocortisone, 300 µg, (*n* = 18) via intranasal administration significantly reduced brain infarct size in both male and female rat pups vs. controls (*n* = 13). No significant difference was seen comparing hydrocortisone, 1000 µg, (*n* = 9) vs. saline (*n* = 13) via intranasal administration. Data are means ± SEM, * *p* < 0.05. Scale bars, 2 mm.

Based on our above findings, we examined whether hydrocortisone post-treatment would also confer neuroprotection in the LPS sensitized neonatal HIE rat model. As demonstrated in Figure 5a, hydrocortisone (30 µg) compared to saline via ICV significantly reduced brain infarct size in male and female rats in the LPS sensitized HIE model (11.6% ± 3.2% vs. 22.2% ± 2.1% for males; 10.9% ± 3.2% vs. 23.5% ± 3.0% for females, *p* < 0.05), respectively.

Hydrocortisone was then given via intranasal administration at two doses (300 µg and 1000 µg) after LPS sensitization and HI injury. The lower hydrocortisone dose of 300 µg was found to significantly decrease brain injury in both genders of neonatal rat pups (10.5% ± 3.7% vs. 25.4% ± 4.2% for males; 12.6% ± 3.1% vs. 27.9% ± 1.3% for females, *p* < 0.05), Figure 5b. Interestingly, the higher hydrocortisone dose of 1000 µg did not confer any protective effects and, although not statistically significant, it seemed to worsen brain infarct size in both male and female pups (25.4% ± 4.2% vs. 30.3% ± 1.5% for males; 27.9% ± 1.3% vs. 32.1% ± 1.3% for females, *p* > 0.05), Figure 5b, further indicating the multiple complex effects of glucocorticoids in brain pathology.

## 3. Discussion

The present study demonstrates a novel finding that hydrocortisone and dexamethasone decrease the vulnerability of the neonatal brain to hypoxic-ischemic encephalopathy (HIE), given 4 h post HI-injury via intracerebroventricular (ICV) injection or via a novel method of intranasal administration, thus providing a potential therapeutic strategy of clinical significance. Hypoxic-ischemic encephalopathy remains to be the leading cause of neonatal brain damage, with a morbidity of two per 1000 live births and approximately six per 1000 births in premature infants [13,14,15]. Hypoxic-ischemic encephalopathy has devastating consequences, including cerebral palsy, seizures, behavioral problems, and neurodevelopmental deficits. Hypoxic-ischemic brain injury results from impaired oxygen flow and glucose delivery to the neonatal brain that ultimately results in cell death and release of inflammatory cytokines. With the limited effects of hypothermia treatment, there is an urgent need to explore effective neuroprotection strategies that may diminish neonatal morbidity and mortality [16].

Several animal models have been used to study perinatal brain injury including non-human primates, sheep, rabbits, and rodents, and all models have contributed to the progress regarding the underlying mechanisms of perinatal hypoxic-ischemic brain injury. Large animal models of sheep allow to access ongoing physiologic parameters in the fetus in response to chronic intrauterine ischemia. Small animal models of rodents have an advantage in the better understanding of biochemical and molecular consequences of perinatal brain injury, and in the assessment of longstanding neuropathologic endpoints and behavioral outcomes. By far the neonatal rat is the most commonly used animal model of perinatal hypoxic-ischemic brain injury, and the main advantage of the rodent model is the extent of its characterization over the years.

Previously, we showed that hypoxia sensitizes the neonatal brain to hypoxic-ischemic encephalopathy (HIE). We demonstrated that hypoxic-ischemic injury decreased glucocorticoid receptor expression. Importantly, treatment with increasing doses of dexamethasone (0.01–0.1 μg), a synthetic glucocorticoid, prior to HI injury provided a neuroprotective effect in rat pup brains [9]. A possible underlying mechanism involves the interaction of glucocorticoids-GR signaling and L-PGDS-PGD2-DP1-pERK mediated pathway [9]. It remains unclear whether glucocorticoids may regulate glutamate and *N*-methyl-d-aspartate (NMDA) receptors in the neonatal brain. In the present study, we modified the HI injury model in seven-day-old neonatal pups, previously used by Vannucci and Vannucci [17] to investigate the effects of dexamethasone and hydrocortisone post-hypoxic insult.

Our initial experiment explored the effectiveness of dexamethasone treatment, via ICV injection and intranasal administration post-HI injury, to treat HIE. Perinatal asphyxia, resulting in HIE, has an extremely narrow therapeutic window and quickly treating newborns is critical [18]. In our study, we show a significant reduction in infarction size with 0.1 μg dexamethasone administration via ICV injection 2 h post-HI event, suggesting that direct neural activation of the GR may play a significant role in perinatal neuroprotection from HI injury. Future studies are needed to further determine glucocorticoid-mediated protection of cell injury and cell death in different regions in the brain, as well as the long-term effect of glucocorticoids in alleviating brain damage.

We next looked at the intranasal dexamethasone administration as a practical clinical technique. Drug delivery to the brain is limited by the blood-brain barrier that separates brain tissues from the systemic circulating system and central interstitial fluid. Intranasal delivery is a powerful, non-invasive method to directly deliver chemicals and peptides to the brain, which is not obstructed by the blood-brain barrier, avoids fast systemic clearance, and limits potential secondary effects. It has been used to deliver chemicals, peptides, oligonucleotides, proteins, and stem cells into the brain. Contradictory to our previous results, we found that increasing doses of intranasal administration of dexamethasone (1 μg, 3 μg, and 30 μg) following the HI event slightly increased the infarction size in the neonatal brain. This may be explained by the U-shaped profile of corticosteroids on the neuronal cells. It was found that when corticosteroid dosing is too high or too low, it can cause excitotoxic injury to the neuronal cells. On the other hand, slightly elevated plasma levels of corticosterone may produce a neuroprotective response that resists excitotoxic damage [7].

Even though dexamethasone is used for chronic lung disease in premature infants, prolonged administration in the rat pup has been shown to negatively affect fetal neurological development and brain weight gain [19,20,21]. This decrease in brain maturation has been found to be associated with neuronal programmed cell death, attenuated maturation, and decreased organization of synaptic connections [22,23]. Hydrocortisone is a better alternative to dexamethasone because at similar dosing, it lacks the adverse side effects [24]. We provide evidence that via an alternative route, through intranasal administration, hydrocortisone has a potential to be used clinically in the treatment of hypoxic-ischemic encephalopathy.

In the present study, we showed that post-HI ICV administration of hydrocortisone significantly decreased the infarction size, suggesting that the activation of the glucocorticoid receptors post hypoxic-ischemia insult may play a neuroprotective role in the neonatal brain. Interestingly, despite the fact that both male and female populations had a decreased infarction size with the administration of hydrocortisone, it is important to note that the female population consistently showed greater infarct size. A recent study found that male neonatal rats were more vulnerable to hypoxic-ischemic brain injury, though the mechanism is still unknown [25]. The present finding showed that the female pups were more susceptible to the hypoxic-ischemic injury and are slightly more resistant to the treatment. Of importance, post-HI intranasal administration of hydrocortisone significantly decreased brain injury in male rat pups, and had a tendency of neuroprotection in females as well. It is possible that increasing sample size may have a significant effect in females due to a somewhat smaller effect observed in female pups. It is interesting that intranasal administration of hydrocortisone, but not dexamethasone, had neuroprotective effects. The reason for this difference is not entirely clear at present, but it may be due to different receptor selectivity and the duration of action of the two glucocorticoids. Dexamethasone is a synthetic, non-hydrolyzable glucocorticoid that selectively acts on glucocorticoid receptor (GR), while hydrocortisone acts on both GR and mineralocorticoid receptor (MR) and is metabolized by 11β-hydroxysteroid dehydrogenases.

We then challenged this model with LPS pre-sensitization to replicate systemic infection contracted before, at, or after the time of birth. Previous studies confirmed that pathogen-induced inflammation increased brain injury in response to hypoxic-ischemia (HI) [26,27,28]. Neonates suffer a higher morbidity and mortality in infection due to an underdeveloped immune system. The innate response in neonates causes a robust, uncontrolled inflammatory response, making neonates more vulnerable to infection [29]. Mechanistically, low dose LPS has been shown to damage the blood-brain barrier in the white matter, as well as increase microglial activation, tumor necrosis factor α (TNF-α) expression, and caspase-3-positive cells [30,31]. In the present study, acute LPS sensitization with the HIE model caused a significant increase in neonatal rat pup mortality that correlated with increases to LPS dosage. Significantly, those pups that were sensitized with LPS had a decreased body weight ratio comparatively to the saline group, possibly due to the increased stress caused by a heightened central and systemic inflammatory response [30].

Without the treatment, the LPS challenge significantly increased neonatal hypoxic-ischemic brain injury, as seen in Figure 4c. In the clinical scenario of neonatal HIE complicated with infection and increased brain injury, therapeutic hypothermia showed no effect of neuroprotective [32], making it a priority to find alternative mechanisms that protect the LPS-sensitized neonatal brain. In our study, LPS-sensitized rat pups given hydrocortisone post-HI event significantly showed a decreased infarction size, confirming hydrocortisone’s ability to provide neuroprotection in a vulnerable infection event.

To be clinically practical in an LPS-sensitized neonatal model, we administered intranasal hydrocortisone post-HI and found that at dose 300 μg, hydrocortisone significantly decreased the infarction size, while at 1000 μg dose, hydrocortisone caused a slight increase in infarction size. This is similar to the results seen in the dexamethasone model, suggesting the U-shaped neuroprotective profile on neuronal cells by glucocorticoids. At 300 μg, hydrocortisone resists excitotoxic injury and, at 1000 μg, hydrocortisone is susceptible to excitotoxic injury, resulting in additional neuronal damage.

A limitation of the present study is that the brain injury was measured by 2,3,5-triphenyltetrazolium chloride (TTC) staining that was unable to accurately separate and quantify the white matter and/or gray matter injury, which are better determined by magnetic resonance imaging (MRI) scans with diffusion-weighted imaging (DWI) and T1-weighted signals. Another limitation is that the functional impact of glucocorticoid-mediated neuroprotection on brain infarct size was not determined. These should be addressed in the future studies. Given that the advent of therapeutic hypothermia offers neuroprotection in HIE but the improvement in outcomes has been modest, a future direction in the field is to explore a combination of adjunct intranasal glucocorticoids with hypothermia in perinatal brain HI injury to improve outcomes and long-term optimization of infant neuroplasticity.

## 4. Materials and Methods

### 4.1. Experimental Animals

Pregnant Sprague-Dawley rats were purchased from Charles River Laboratories (Portage, MI, USA). Animals were allowed to give birth and were then kept with their pups in a room maintained at 24 °C with a 12-h light/dark cycle, and provided ad libitum access to normal rat chow and filtered water. Treatment groups were based on litters, with pups of both sexes being subjected to HIE treatment and being randomized to receive either saline (placebo) or treatment with dexamethasone, hydrocortisone, LPS sensitization, or LPS sensitization with hydrocortisone treatment as described below. All procedures and protocols were approved by the Institutional Animal Care and Use Committee of Loma Linda University (IACUC# 8160017, approved on 9 May 2016) and followed the guidelines by the National Institutes of Health Guide for the Care and Use of Laboratory Animals.

### 4.2. Neonatal Hypoxic–Ischemic Encephalopathy (HIE) Rat Model

The Rice–Vannucci rat model of unilateral common carotid artery ligation followed by 8% FiO_2_ treatment has been widely used in studies of neonatal brain damage, and P7 rats were used in this study to simulate term newborn infants [11,33]. A modified Rice–Vannucci model was used in this study, and in brief, P7 pups were anesthetized with 2% isoflurane in oxygen. The adequacy of anesthesia was determined by the loss of a pedal withdrawal reflex and any other reaction from the animal in response to pinching the toe, tail, or ear of the animal. A small incision was made in the right side of neck where the right common carotid artery was exposed, double ligated with silk surgical sutures, and then transected between the sutures. The incision was then sutured closed, and after recovery for one hour, pups were treated with 8% FiO_2_. Hypoxia exposure time varied slightly depending on different study groups with 120–150 min for intact pups, 50 min for LPS acutely challenged pups and 100 min for LPS chronically challenged pups, Figure 1.

### 4.3. Lipopolysaccharides (LPS) Sensitization of Neonatal HIE Rat Model

Lipopolysaccharides (LPS) (SIGMA-ALDRICH; catalog #L4524; lipopolysaccharides from *Escherichia coli* 055:B5, purified by ion-exchange chromatography, TLR ligand tested) were administered before HIE in acute or chronic challenged manner, respectively. Acute sensitization: LPS (0.3 mg/kg, 0.1 mg/kg, or 0.05 mg/kg) was given via intraperitoneal (IP) injection 4 h before HI injury, hypoxia exposure time was 50 min. Chronic sensitization: LPS (0.05 mg/kg) was given via IP injection on days P3 and P5, with HI injury on P7 (hypoxia exposure time 100 min). All other procedures are the same as the previously described neonatal rat HIE model. An equal volume of IP saline served as the vehicle control.

### 4.4. Intracerebroventricular (ICV) Injection

For the ICV treatment groups, the ICV injection was preformed 2 h following the HI injury as has been previously described [34]. Pups were anesthetized with 2% isoflurane and fixed on a stereotaxic apparatus (Stoelting, Wood Dale, IL, USA). An incision was made on the skull surface and the bregma was exposed. Dexamethasone or hydrocortisone was dissolved in saline and 2 μL was injected at a rate of 1 μL/min with a 10 μL syringe (Stoelting, Wood Dale, IL, USA) in the right hemisphere following the coordinates relative to the bregma: 2 mm inferior, 1.5 mm lateral, and 3.0 mm below the skull surface. The control group was injected with 2 μL of saline alone. The injection lasted 2 min and the needle was kept inserted for an additional 5 min before its removal. The incision was sutured following the procedure, the anesthesia was withdrawn, and the pups were returned to their dams.

### 4.5. Intranasal Treatment

For the intranasal treatment groups, the intranasal instillation was performed 2 h after the HI injury as has previously been described [10]. Briefly, rat pups were placed in a supine position and sedated with 2% isoflurane anesthesia. Dexamethasone and hydrocortisone were both dissolved in saline and, depending on the concentration, 2.5–7.5 μL of the drug was administered alternately into the right and left nostril with an applicator pipette and corresponding tips. Equal volumes of saline were given intranasally to the control groups. Each nostril instillation was given over 5 min, for a total period of 10 min after which the anesthesia was withdrawn and the pups were returned to their dams.

### 4.6. Measurement of Infarction Size

Pups were euthanized 48 h after the HI treatment. Coronal slices of the brain (2-mm thick) were cut and immersed in a 2% solution of 2,3,5-triphenyltetrazolium chloride monohydrate for 5 min at 37 °C, followed by fixation with 10% formaldehyde overnight [35]. Both sides of each slice were photographed separately. The infarction area was analyzed by Image J software (Version 1.40; National Institutes of Health, Bethesda, MD, USA), summed for each brain, and expressed as a percentage of the whole brain.

### 4.7. Statistical Analysis

Data is expressed as mean ± SEM. Experimental number (*n*) represents pups from multiple dams. Data was assessed by one way analysis of variance (ANOVA) followed by Neuman-Keuls post-hoc testing or Student’s *t*-test, where appropriate, using the Graph-Pad Prism software (GraphPad Software Version 4, San Diego, CA, USA). For all comparisons, *p* < 0.05 indicated statistical significance.

## 5. Conclusions

The research thus far has been unclear on whether glucocorticoids provide a beneficial effect of neuroprotection in neonatal HI injury. The present study provides experimental evidence that glucocorticoids given after HI injury have a promising therapeutic effect of neuroprotection in a rat model of neonatal HIE. Of critical importance, intranasal administration of hydrocortisone has been shown to be effective in decreasing brain injury in neonatal HIE complicated with systemic infection, which commonly occurs in infants who suffer from HI brain injury. Future studies are needed to further investigate the mechanisms underlying glucocorticoid-mediated neuroprotection in the neonatal brain, and to explore the possibility of combined treatment of therapeutic hypothermia and glucocorticoids in neonatal HIE.
